# Brain More Resistant to Energy Restriction Than Body: A Systematic Review

**DOI:** 10.3389/fnins.2021.639617

**Published:** 2021-02-09

**Authors:** Marie Sprengell, Britta Kubera, Achim Peters

**Affiliations:** Center of Brain, Behavior and Metabolism (CBBM), University of Lübeck, Lübeck, Germany

**Keywords:** brain energy metabolism, body weight, caloric restriction, high-energy phosphates, selfish brain theory, systematic review

## Abstract

The gluco-lipostatic theory and its modern variants assume that blood glucose and energy stores are controlled in closed-loop feedback processes. The Selfish Brain theory is based on the same assumptions, but additionally postulates that the brain, as an independent energy compartment, self-regulates its energy concentration with the highest priority. In some clinical situations these two theories make opposite predictions. To investigate one of these situations, namely caloric restriction, we formulated a hypothesis which, if confirmed, would match the predictions of the Selfish Brain theory—but not those of the gluco-lipostatic theory. Hypothesis: Calorie restriction causes minor mass (energy) changes in the brain as opposed to major changes in the body. We conducted a systematic review of caloric-restriction studies to test whether or not the evaluated studies confirmed this hypothesis. We identified 3,157 records, screened 2,804 works by title or abstract, and analyzed 232 by full text. According to strict selection criteria (set out in our PROSPERO preregistration, complying with PRISMA guidelines, and the pre-defined hypothesis-decision algorithm), 8 papers provided enough information to decide on the hypothesis: In animals, high-energy phosphates were measured by ^31^P-nuclear magnetic resonance, and organ and total body weights were measured by scales, while in humans organ sizes were determined by magnetic resonance imaging. All 8 decidable papers confirmed the hypothesis, none spoke against it. The evidence presented here clearly shows that the most accurate predictions are possible with a theory that regards the brain as independently self-regulating and as occupying a primary position in a hierarchically organized energy metabolism.

## Introduction

In modern obesity research, most scientists support the theoretical notion that food intake is regulated by closed-loop feedback processes. Already in the 1950s, Mayer came up with the idea that blood glucose is the main regulated quantity in human energy metabolism (Mayer, 1953). Accordingly, a low blood glucose concentration stimulates food intake, which in turn restores a normal blood glucose level. Mayer's framework was called glucostatic theory. At the same time, Kennedy made a counterproposal (Kennedy, 1953). He believed that body weight was the regulated quantity. He postulated that a signal from the body energy stores controls food intake. Yet he could not name this signal. Kennedy's lipostatic theory gained momentum in the 1990s when leptin was identified as the substance from adipose tissue that he had suspected (Zhang et al., 1994).

From today's perspective, Mayer and Kennedy were essentially right, and there are many modern variants of the gluco-lipostatic theory (Chaput and Tremblay, 2009; Schwartz et al., 2017). All these variants have in common that the brain is regarded as passively supplied from the blood. Proponents of these variants see, from a control theory perspective, two factors causing obesity: first, a stimulatory input into the feedback loop, e.g., hedonic eating (Lowe and Butryn, 2007), or second, a disruption in the feedback loop, such as lack of appetite suppression due to leptin resistance, central insulin resistance or the failure of gastrointestinal hormones (Chaudhri et al., 2006; Myers et al., 2008; Kullmann et al., 2020).

At the beginning of the millennium a rival theory was formulated, the Selfish Brain theory (Peters et al., 2004). This framework expanded the gluco-lipostatic theory by a new compartment, the brain as an independent, self-regulating organ delimited by the blood-brain barrier. The theory postulated a primacy that in case of malnutrition or stress there is a vital ability of the human brain, namely to give priority to the own energy metabolism. The postulate was supported by evidence from the 1990s, which showed that when a neuron fires and needs more energy to do so, it pulls glucose from the blood via the astrocytes (Pellerin and Magistretti, 1994). Against this background, the brain turns into an active part in energy metabolism according to the principle of “energy on demand” (Magistretti et al., 1999). The Selfish Brain theory took into account additional—previously unnamed—causes of obesity, such as the brain needing less energy and demanding less energy from the body (e.g., by stress habituation or central suppressant drugs)—so that energy accumulates in the body (Peters and McEwen, 2015; Kuzawa and Blair, 2019). As in economic supply chains, where goods stay on the shelves when customers don't buy, energy accumulates in adipose tissue when the brain demands less energy (Peters and Langemann, 2009).

From an epistemological point of view, however, the introduction of an entity such as the selfish brain also has downsides. Because a good theory should be as simple as possible and make the most accurate predictions (Gilad-Bachrach et al., 2003). Such an extension with the brain as an independent self-regulated compartment makes the Selfish Brain theory more complex than the gluco-lipostatic theory.

Given its higher complexity, the Selfish Brain theory has to face the question whether it can actually make more accurate predictions than the gluco-lipostatic theory. To our knowledge, both theoretical approaches can explain most of the available experimental data. However, in one crucial point the two theories make opposite predictions. On this point, we formulated a hypothesis which, in case of its confirmation, would match the predictions of the Selfish Brain theory—but not those of the simpler gluco-lipostatic theory:

*Hypothesis: Calorie restriction causes minor mass (energy) changes in the brain as opposed to major changes in the body*.

To this end, we conducted a systematic review to test whether the caloric restriction studies found actually confirm this hypothesis or not.

## Materials and Methods

Prior to our work on this systematic review, our protocol was registered on Prospero on 30th of January 2020, and an updated version was published on 28th of September 2020 (International prospective register of systematic reviews; CRD42020156816). We complied with the PRISMA (preferred reporting items for systematic reviews and meta-analyses) guidelines for systematic reviews of interventions (Moher et al., 2009).

### Search Strategies

We conducted a systematic search of the literature to identify studies in humans and other mammals that focused clearly on how energy restriction affects energy states in the brain and body. The search strategies were developed by one reviewer and discussed with two other reviewers. The databases of MEDLINE and BIOSIS Previews were searched from their inception to 30 March 2020, using a combination of keywords and in case of the first database MeSH terms. Thereby, keywords were identified based on previous knowledge, initial research, and a thesaurus. The full MEDLINE and BIOSIS search strategies are provided in the Supplementary Information. Briefly, the search strategies included terms relating to the intervention (caloric restriction), to outcomes (brain and peripheral energy states) and to methodical approach (experimental study), combined by the Boolean operator AND. Synonyms for terms were combined with the operator OR.

### Study Selection

The following criteria were used to include or exclude articles for our systematic review. Only studies published in English or German were included. Only original full research paper were included. Regarding humans, interventional studies were included, either clinical randomized controlled trials (with a non-exposed control group) or standardized laboratory experiments (within-subject-design or between-subject-design). Regarding animals, interventional studies as standardized laboratory experiments (with a non-exposed control group) were included (within-subject-design or between-subject-design). We included studies with healthy humans or other mammalian species, regardless of gender or physical phenotype, with subjects who have neither a known disease nor a drug regimen proven to interfere with energy metabolism (see Supplementary Information for details). We did not include trials in pregnant individuals or fetuses, nor in ovariectomized or genetically modified individuals with altered energy metabolism. We have not included studies in which caloric restriction was induced by an increase in litter size, nor those in which more than one intervention was performed. Particular attention is paid to the distinction between the central and peripheral energy states. Only studies that map both compartments allow us to make comparisons and draw conclusions about the energy distribution between brain and body. Therefore, we only included studies that examined the brain as a whole, or at least most of it (and not just specific regions, such as the hippocampus). And we only included studies that provided information about both central and peripheral energy states.

**Figure 1:**
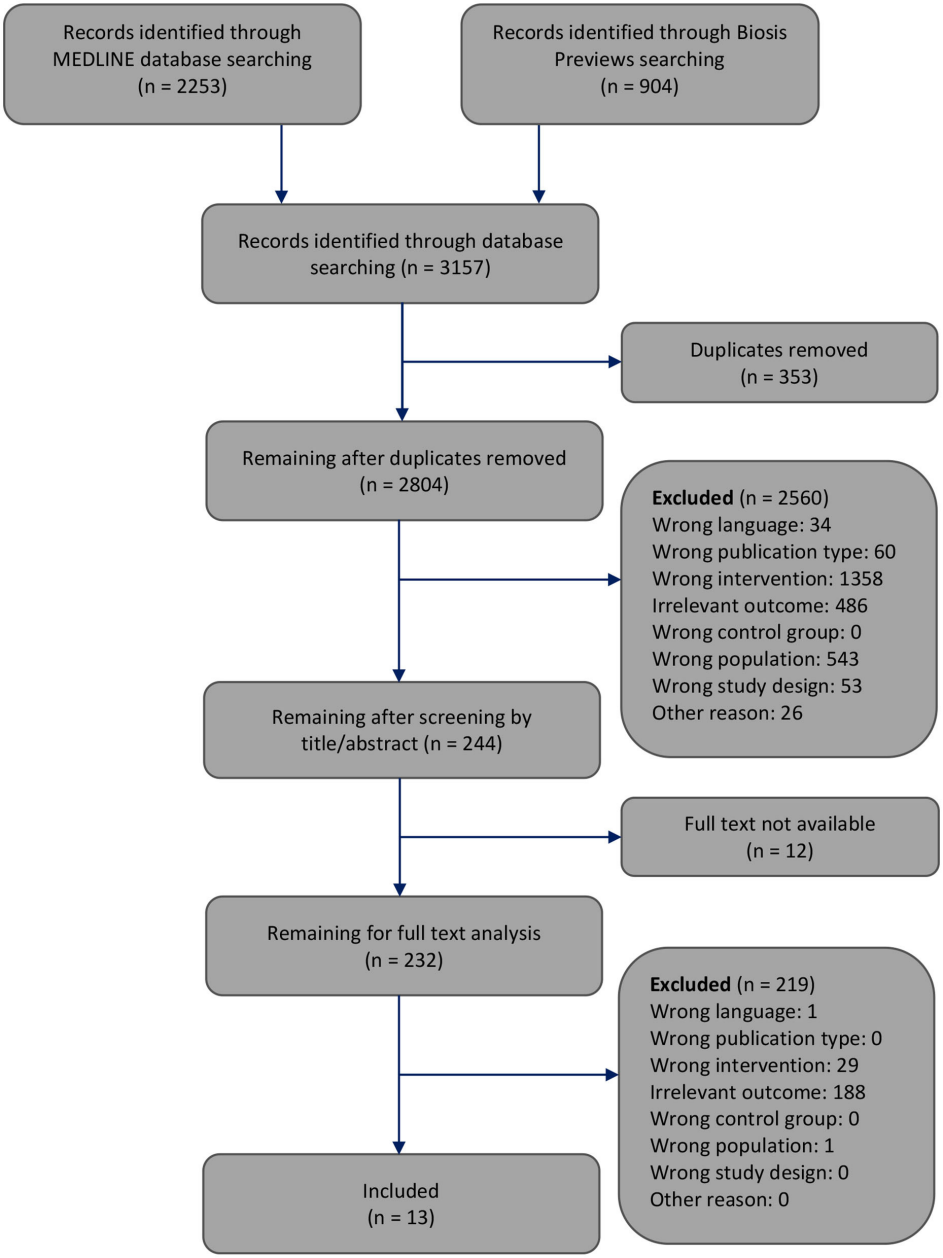
Flowchart through different phases of the systematic review modified according to Moher's publication (Moher et al., 2009).

The selection of the articles was performed in two steps. At each step, reasons for excluding articles were reported (Figure 1). First, one reviewer screened the article titles or abstracts against the inclusion and exclusion criteria. This first step of article selection was checked by another reviewer. When a discrepancy occurred regarding the inclusion or exclusion of an article, the two reviewers discussed it until agreement was reached. Otherwise, disagreements were resolved by consulting the third reviewer. Second, two reviewers independently selected the remaining articles by analyzing the full text. Again, disagreements regarding the inclusion or exclusion of an article were resolved by discussion among each other or, if necessary, by consultation with the third reviewer. In this step of full text analysis, most studies (*n* = 188) were excluded since these studies provided outcomes of only the central nervous energy state or only the peripheral energy state, but not both.

### Data Extraction

Data from all of the 13 included studies were extracted by one reviewer, independently checked by two other reviewers, and tabulated alphabetically. Where results were reported for subgroups within the same article, we extracted the data separately for these subgroups. We recorded the population, sample size, kind of intervention and duration, statistical test applied, as well as kind of body and brain outcomes (i.e., ATP or mass). Whenever possible (which was the case in 10 of 13 papers), we also calculated the percentage changes in body and brain induced by the intervention.

### Risk of Bias Assessment

One reviewer assessed the risk of bias of the included studies using the SYRCLE‘s tool for non-human studies (Hooijmans et al., 2014) or the Cochrane Collaboration's tool updated in 2011 (Higgins et al., 2011) for studies conducted in humans. The results were independently checked by two other reviewers. All differences were clarified by discussion.

### Hypothesis Decision

**Figure 2:**
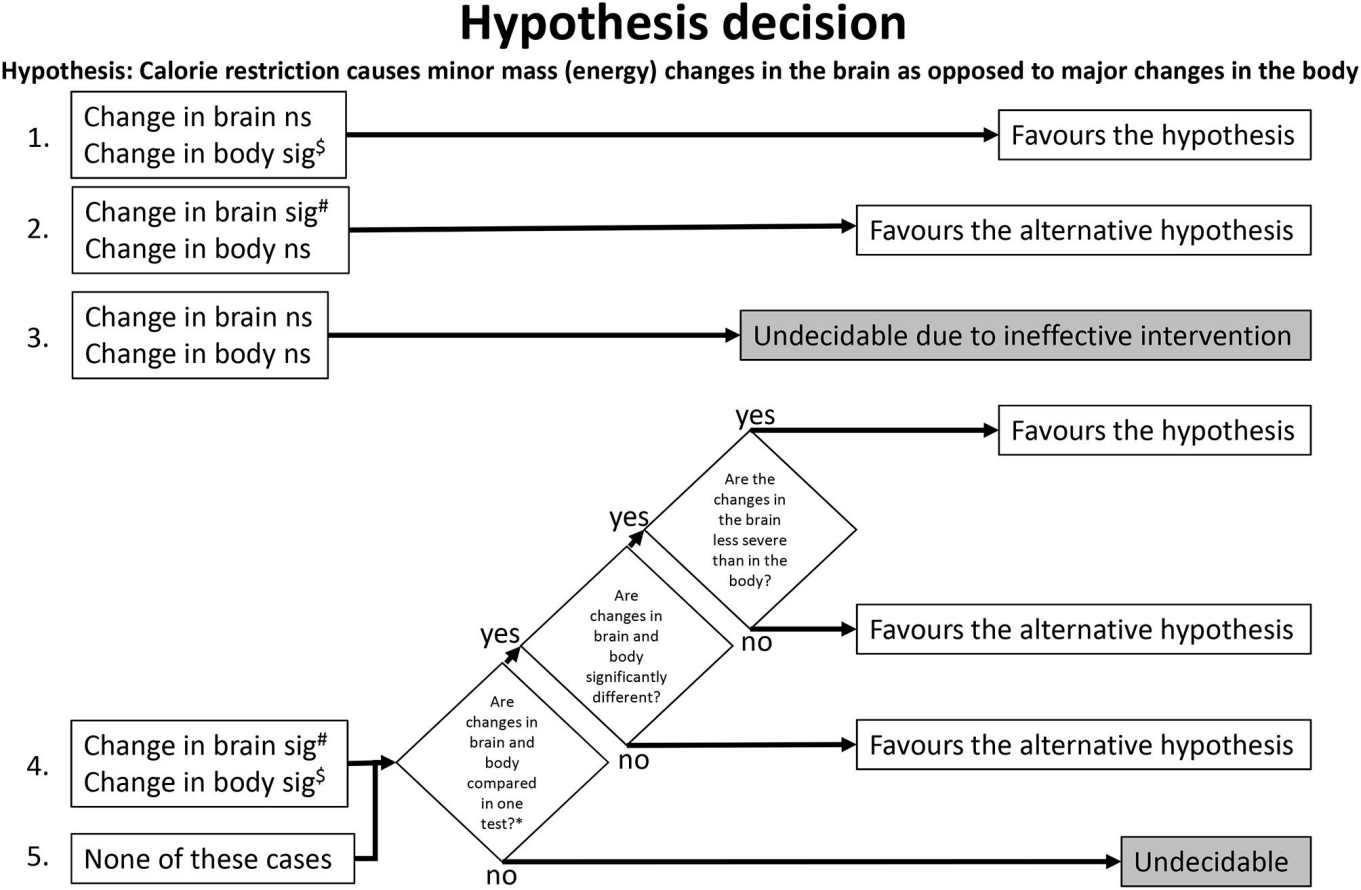
Algorithm for hypothesis decision. The numbers on the left indicate where the algorithm path begins for a given study. ^$^Overriding exception (unlikely to occur): A significant (sig) increase in the body favors the alternative hypothesis. ^#^Overriding exception (unlikely to occur): A significant increase in the brain favors the hypothesis. *e.g., testing relative brain changes (like change of brain mass/body mass). ns, not significant.

We have predefined the following algorithm (Figure 2) for later use in the review process, when the step of hypothesis decision is due. This algorithm included all combinations of brain and body findings and their statistical analysis. We used the algorithm to clarify whether a study allows a hypothesis decision (decidable study) or not (undecidable study). If decidable, we used the algorithm to check whether the study favors the hypothesis or the alternative hypothesis. Hypothesis decision was conducted by two reviewers independently and agreed by a third reviewer.

## Results

The systematic search of the literature generated 3,157 articles, which were processed as summarized in Figure 1. Two thousand eight hundred four works were screened by title or abstract, and 232 articles were analyzed by full text. We identified 13 studies that met all inclusion criteria and focused on how energy restriction affects the central and peripheral energy states (Schärer, 1977; Villeneuve et al., 1977; Goodman and Ruderman, 1980; Harris et al., 1984; Ocken and Grunewald, 1988; Bodoky et al., 1995; Wolff et al., 1999; Dubnov et al., 2000; Greenberg and Boozer, 2000; Ostrowski et al., 2006; Peters et al., 2011; Hisatomi et al., 2013; Ryzhavskii et al., 2019).

### Characteristics of Included Studies

Table 1 provides details of the 13 included studies. Eight studies examined rats (Schärer, 1977; Villeneuve et al., 1977; Goodman and Ruderman, 1980; Harris et al., 1984; Ocken and Grunewald, 1988; Bodoky et al., 1995; Greenberg and Boozer, 2000; Ryzhavskii et al., 2019) and three studies investigated mice (Wolff et al., 1999; Dubnov et al., 2000; Hisatomi et al., 2013). One study was conducted in sand gazelles (Ostrowski et al., 2006). Only one study reported on humans, women in particular (Peters et al., 2011). The sample sizes varied between 10 and 150.

**Table 1:** Characteristics and results of included studies.

**Study**	**Population**	**Sample size**	**Intervention**	**Duration**	**Statistical test**	**Body outcome**	**Brain outcome**	**Difference in body outcome**	**Difference in brain outcome**
Bodoky et al. (1995)	Fischer 344 rats, male	Exp.: 8 Con.: 8	Complete food deprivation; water *ad libitum*	4 days	ANOVA multi-comparison	Liver ATP/PME ratio^*^ Liver ATP/PDE ratio^*^	ATP/PME ratio^ns^ ATP/PDE ratio^ns^	Liver ATP/PME ratio: −42.59% Liver ATP/PDE ratio: −33.89%	Brain ATP/PME ratio: −6%^a^ Brain ATP/PDE ratio: −2%^a^
Dubnov et al. (2000)	Sabra mice, female	Exp.: 12 Con.: 12	Food restriction to 60% of daily requirement	40 days	Two-tailed *t*-test	Body weight^*****^	Abs. brain weight^ns^	−34.09%	−4.58%
Goodman and Ruderman (1980)	Sprague-Dawley rats, male (Exp._1_: 8-week-old, 200 g; Exp._2_: 16-week-old, 450–500 g; Exp._3_: 16-week-old, obese)	Exp._1_: 9 × 6^b^ Exp._2_: 7 × 6^b^ Exp._3_: 9 × 6^b^	Complete food deprivation; water *ad libitum*	Exp._1_: 5 days Exp._2_: 10 days Exp._3_: 20 days	*t*-test	Exp._1_: Body weight^**^ Exp._2_: Body weight^**^ Exp._3_: Body weight^**^	Abs. brain weight^c^	Exp._1_: −37.6% Exp._2_: −31.07% Exp._3_: −38.52%	Quote: “well-maintained”
Greenberg and Boozer (2000)	Fischer 344 rats, male	Exp.: 10 Con.: 9	Gradual food restriction to 60% of that of controls	~17 months	ANOVA with Tamhane's *post-hoc* test	Body weight^*^	Abs. brain weight^ns^	−39.65%	−2.4%
Harris et al. (1984)	Wistar rats, male and female (Exp.: undernourished a + b; Con.: well-nourished a + b)	Exp.(m): 14 Con.(m): 14 Exp.(f): 14 Con.(f): 14	Food restricted to reduce body weight by 1/3	4 weeks (Trial 2)	Analysis of variance	Body weight^c^	Abs. brain weight^c^ Rel. brain weight^c^	m: −38%^a^ f: −37%^a^	Abs. brain weight (m): −2%^a^ Abs. brain weight (f): −1%^a^
Hisatomi et al. (2013)	C57BL/6J mice, male	Exp.: 5 Con.: 5	Complete food deprivation; water *ad libitum*	3 days	*t*-test	Body weight^c^	Abs. brain weight^c^	No data	No data
Ocken and Grunewald (1988)	Wistar weanling rats, male	Exp.: 8/9 Con.: 8/9	Complete food deprivation every other day; water *ad libitum*	4 weeks	Analysis of variance	M. gastrocnemius^*^ M. vastus^*^	Abs. brain weight^*^	M. gastrocnemius: −27.65% M. vastus: −29.28%	−8.59%
Ostrowski et al. (2006)	Sand Gazelles, male	Exp.: 6 Con.: 6	Gradual food and water restriction by 15% every 3 weeks to 30–40% of that of controls	4 months	Two-tailed *t*-test	Body weight^*^ Muscle^****^	Abs. brain weight^ns^ Rel. brain weight^ns^	Body weight: No data Muscle: −26.61%	Abs. brain weight: −6.14%
Peters et al. (2011)	Caucasian women	Exp: 42^d^ Con: 52	Low-calorie diet (800–1,000 kcal/day)	12.7 weeks	Depended *t*-test, Wilcoxon non-parametric test	Body weight^***^ Liver weight^**^^e^	Abs. brain weight^ns^	Body weight: −11.13% Liver weight: −4,72%	+0.31%
Ryzhavskii et al. (2019)	Rats (strain not specified), 1 month old, male and female	Exp: 9 Con.: 8	Food restriction to 33% of that of controls	15 days (series 1)	*t*-test	Body weight^*^	Abs. brain weight^*^ Relative brain weight^*^	−35.62%	−5.3%
Schärer (1977)	Growing Füllinsdorf Albino rats, male	Exp._1_: 12 Exp._2_: 12 Exp._3_: 12 Con.: 12	Exp._1_: food restriction to 62% of that of controls Exp._2_: food restriction to 66% of that of controls Exp._3_: Gradual food restriction to 38% of that of controls	Exp._1_: 13 weeks Exp._2_: 9 weeks Exp._3_: 4 weeks	*t*-test	Exp._1_: Body weight^f^ Exp._2_: Body weight^f^ Exp._3_: Body weight^f^	Exp._1_: Abs. brain weight^***^ Exp._2_: Abs. brain weight^***^ Exp._3_: Abs. brain weight^***^ Exp._1_: Rel. brain weight^***^ Exp._2_: Rel. brain weight^***^ Exp._3_: Rel. brain weight^***^	Exp._1_: −33.61% Exp._2_: −31.42% Exp._3_: −26.5%	Exp._1_ abs. brain weight: −7.4% Exp._2_ abs. brain weight: −6.55% Exp._3_ abs. brain weight: −6.28%
Villeneuve et al. (1977)	Wistar SPF rats, male and female	Exp._1_ (m): 6 Con._1_ (m): 6 Exp._2_ (f): 6 Con._2_ (f): 6	Food restriction to 50% of that of controls	4 weeks	Unclear	Body weight^f^	Rel. brain weight^*^	m: −25.77% w: −23.41%	No data
Wolff et al. (1999)	Agouti A/a mice, female	Exp.: 39 Con.: 35	Gradual food restriction to 70% of that of controls	57 weeks	Analysis of variance	Body weight^c,g^	Abs. brain weight^c,h^	≥-26.15%^c^	−6.81%

*Only results from study arms that fulfill our inclusion criteria are listed. Quotes are from original papers. Exp., experimental group; Con., control group; m, male; f, female; PME, phosphomonoester; PDE, phosphodiester; Abs., absolute; Rel., relative*.

* *p < 0.05*,

** *p < 0.01*,

*** *p < 0.001*,

**** *p < 0.0001*,

***** *p < 0.00001, ns, not significant*.

a *The data was only displayed graphically in the publication; values obtained from the graph by reviewers*.

b *In the first experiment, 8-week-old rats were studied in 9 groups of 6 animals each; 5 groups at baseline and 4 groups after caloric restriction for 1, 2, 4, and 5 days. In the second experiment, 16-week old rats were studied in 7 groups of 6 animals each; 4 groups at baseline and 3 groups after caloric restriction for 2, 5, and 10 days. In the third experiment, 16-week-old obese rats were studied in 9 groups of 6 animals each; 5 groups at baseline and 4 groups after caloric restriction for 2, 5, 10, and 20 days. Outcomes in all experiments were measured before and after the intervention in each group*.

c *No data about significance*.

d *Study comparing organ weights (between lean and obese subjects) and organ weight changes in obese subjects (under caloric restriction). There was no detectable difference in brain weight between lean and obese subjects; the obese subjects were calorie-restricted and showed the changes in brain and body outcomes reported here*.

e *Liver size was determined by MRI, as was brain size*.

f *Not clear whether the statistical test was significant or not; the absence of a corresponding footnote for the body weight value in the table speaks against significance; the large difference between the body weight mean values at very small standard deviations speaks for significance*.

g *Brain and body weighted at different times*.

h *Brain weights of CR and AL mice were tested for significance only for Mottled yellow A^vy^/A and agouti A/a mice together, not separately*.

All 13 included studies provided details on how the calorie restriction was implemented. A considerable spectrum was found: The duration of the calorie restriction ranged from 3 days to 17 months. Three studies used complete food deprivation (Goodman and Ruderman, 1980; Bodoky et al., 1995; Hisatomi et al., 2013), others used an intermittent fasting regimen (Ocken and Grunewald, 1988). Food was restricted to about 30–40% (Ostrowski et al., 2006; Ryzhavskii et al., 2019), to 50% (Villeneuve et al., 1977), to 60% (Greenberg and Boozer, 2000), and to about 70% of that of controls (Wolff et al., 1999). In other studies, food was restricted to reduce body weight by 33% (Harris et al., 1984), or to reduce it to 60% of daily requirements (Dubnov et al., 2000). In a further study, experiments were conducted with a food restriction at 60–70% of the controls, compared to a gradual food restriction to 38% (Schärer, 1977). The only study conducted in humans used a low-calorie diet (800–1,000 kcal/day) (Peters et al., 2011).

All animal studies used autopsies to assess brain and body outcomes. The human study bypassed autopsy by measuring organ sizes by magnetic resonance imaging (MRI) (Peters et al., 2011). Most studies reported intervention-induced changes in body weight and absolute brain weight (Goodman and Ruderman, 1980; Wolff et al., 1999; Dubnov et al., 2000; Greenberg and Boozer, 2000; Peters et al., 2011; Hisatomi et al., 2013). Four studies additionally reported the relative brain weight (Schärer, 1977; Harris et al., 1984; Ostrowski et al., 2006; Ryzhavskii et al., 2019). Others reported muscle weight and absolute brain weight (Ocken and Grunewald, 1988), or the combination of body weight and relative brain weight (Villeneuve et al., 1977). One study reported on high energy phosphates, i.e., the ratio of ATP/PDE (adenosine triphosphate/phosphodiester) in liver and brain (Bodoky et al., 1995). Four of the 13 included studies provided incomplete data or statistical information on their results (Goodman and Ruderman, 1980; Harris et al., 1984; Wolff et al., 1999, Hisatomi et al., 2013).

In the thirteen included studies, the changes in brain mass or energy concentration ranged from +0.3 to −8.6%, while the changes in body mass or rather energy ranged from −11.1 to −40.0%.

### Risk of Bias Assessment

To assess the risk of bias of non-human studies the SYRCLE‘s tool was used (Hooijmans et al., 2014). To assess the risk of bias of human studies the Cochrane Collaboration's tool updated in 2011 was used (Higgins et al., 2011). The seven items of the latter tool were integrated in the abovementioned tool. Table 2 provides the risk of bias assessment for all 13 included studies. Five studies (41.7% of the included studies) reported randomization. In all included studies, the baseline characteristics of the intervention and control groups were similar, indicating a low risk of bias. Blinding of outcome assessors was not possible in animal studies, since restricted food supply, weight changes and altered behavior (starvation-induced locomotion) was openly visible to animal keepers. In the human study, where brain size was assessed by MRI, blinding of the outcome assessor was theoretically possible, but not explicitly reported in the publication (Peters et al., 2011). All studies were classified as low risk for attrition bias. Ten studies (76.9%) were rated low risk for reporting bias. Only one study was rated high risk of other sources of bias as the conditions in which animals were kept differed between experimental and control groups; moreover, in this study, body mass was measured earlier than indicated in the protocol (Wolff et al., 1999).

**Table 2:** Risk of bias assessment.

	**Random sequence generation**	**Baseline characteristics**	**Allocation concealment**	**Blinding of outcome assessors**	**Addressing of incomplete outcome data**	**Selective outcome reporting**	**Other sources of bias**
Bodoky et al. (1995)	+	+	+^a^	n.a.	+^b^	+^c^	+^d^
Dubnov et al. (2000)	?	+	?	n.a.	+^e^	+	+^d^
Goodman and Ruderman (1980)	?	+	?	n.a.	+^e^	–^f^	+^d^
Greenberg and Boozer (2000)	?	+	?	n.a.	+^g^	+	+^d^
Harris et al. (1984)	+	+	+^a^	n.a.	+^b^	–^h^	+^d^
Hisatomi et al. (2013)	?	+	?	n.a.	+^b^	–^i^	+^d^
Ocken and Grunewald (1988)	+	+	+^a^	n.a.	+^e^	+^j^	+^d^
Ostrowski et al. (2006)	+	+	+^a^	n.a.	+^b^	+^k^	+^d^
Peters et al. (2011)	n.a.	n.a.	n.a.	?^l^	+	+	+^d^
Ryzhavskii et al. (2019)	?	+^m^	?	n.a.	+^e^	+	+^d^
Schärer (1977)	+	+	+^a^	n.a.	+^b^	+	+^d^
Villeneuve et al. (1977)	?	+	?	n.a.	+^b^	+^n^	+^d^
Wolff et al. (1999)	?	+	?	n.a.	+^e^	+	–^o^

*The risk of bias was rated as either low “+” or high “–,” given sufficient information was available, otherwise it was marked “?”. Not every item is applicable to each study intervention (“n.a.”). For studies with a within-subject-brain-to-body comparison the item on randomization is not applicable (“n.a.”). For two items of the SRYCLE tool, i.e., random placement and random result assessment, no information was available for any of the studies examined here, so that all of them were rated “?”. A further item of this tool, namely the blinding of personnel (participants), is not applicable for calorie restriction studies, since weight changes, restricted food supply and altered behavior (starvation-induced locomotion) are openly visible to animal keepers*.

a *Given first, documented randomization without reference to an open randomization scheme and second, similar baseline characteristics, there is already a high “amount of information” (i.e., mutual information) about the risk of bias in terms of allocation concealment. In this case, the risk of bias regarding the randomization procedure can be considered low*.

b *No dropouts*.

c *31P-Nuclear Magnetic Resonance study (NMR), which reported and statistically compared all relevant high-energy phosphate results in brain and liver. Total body weight, as opposed to high-energy phosphate like ATP and ADP, was not a primary outcome in this NMR study. That changes in total body weight were only reported in a descriptive way, probably only indicates a low risk of bias regarding changes in organ-specific high-energy-phosphates during fasting*.

d *No evidence of critical housing conditions, problems associated with study design, or conflicts of interest*.

e *No evidence for dropouts*.

f *The authors stated “the weights of the adrenals, brain, and testes were well maintained,” but they did not show this data. We classified the risk of bias due to incomplete outcome data reporting as high*.

g *Dropouts, reason not reported. Of planned 10 fasting and 10 control rats, 10 fasting and 9 control rats were actually examined and reported. Given the large differences in effects that have been demonstrated for brain and body outcomes, one drop-out probably indicates only a low risk of bias when studying changes in brain and body weights under caloric restriction*.

h *The authors stated “undernutrition affects some organs severely (e.g., the liver) while others (e.g., the brain) lose little or no weight over the period of food deprivation,” and they actually displayed brain and body weight data graphically, but without the result of statistical comparison. Absolute and relative brain weight were shown for only one of two trials. Statistical tests were performed, but the result was not shown in every case*.

i *The authors stated “there were no changes in the wet weight of the brain between groups,” but they did not show data on brain weight and body weight changes, nor statistical test results on these data*.

j *Study on the early development of the brain and other organs during undernutrition, which reported and statistically compared all relevant outcomes, i.e., weights of brain, heart, liver, kidneys, tibia, femur, and gastrocnemius and vastus muscle. In 4-week old rats, total body weight was measured before randomization, and weight gain was determined after 4 weeks of fasting in the intervention group and the control group. All these values were shown. That total body weight was not shown in the groups immediately after randomization probably indicates only a low risk of bias regarding organ growth under early undernutrition*.

k *Study on the development of the brain and other organs under food restrictions, which reported and statistically compared all relevant outcomes listed in the methods section (weights of brain, heart, liver, kidney, rumen, intestine, skin, and left muscles of the M. fibularis tertius). Total body weight was determined at baseline, after 4.5 days and after 4 months of fasting and statistically compared to the control group (ANOVA for repeated measures, time × intervention) and statistical results were shown [ANOVA F_2,15_ = 33.9, P < 0.0001 plus post-hoc tests Newman-Keuls, P < 0.05]. That the early and late changes in total body weight in the control group were not explicitly shown probably only indicates a low risk of bias regarding organ size changes during food restriction*.

l *Blinding in MRI brain volume assessment is not explicitly reported, but would have been theoretically possible*.

m *Strain not specified, but baseline body weights similar*.

n *Relative brain weight shown instead of the absolute brain weight*.

o *Calorie-restricted mice kept individually, ad libitum-fed mice kept in pairs. Body weight was measured earlier than indicated in the protocol*.

### Hypothesis Decision

Here we used the predefined hypothesis-decision algorithm described in the Methods section (Figure 2). The algorithm indicated that out of the 13 included studies, 8 studies were decision-ready, and 5 were undecidable. Of the 8 studies where the decision was pending, 8 supported the hypothesis and none supported the alternative hypothesis (Table 3).

**Table 3:** Hypothesis decision.

**Study**	**Change in brain**	**Change in body**	**Changes in brain and body compared in one test?**	**Algorithm path used for the decision**	**Hypothesis decision (either decidable or undecidable)**	**In favor of the hypothesis (either yes or no)**
Bodoky et al. (1995)	n.s.	↓		1	Decidable	yes
Dubnov et al. (2000)	n.s.	↓		1	Decidable	yes
Goodman and Ruderman (1980)	Data not shown	↓	No	5	Undecidable	
Greenberg and Boozer (2000)	n.s.	↓		1	Decidable	yes
Harris et al. (1984)	Not tested	Not tested	No	5	Undecidable	
Hisatomi et al. (2013)	Not tested	Not tested	No	5	Undecidable	
Ocken and Grunewald (1988)	↓	↓	No	4	Undecidable	
Ostrowski et al. (2006)	n.s.	↓		1	Decidable	yes
Peters et al. (2011)	n.s.	↓		1	Decidable	yes
Ryzhavskii et al. (2019)	↓	↓	Yes	4	Decidable	yes
Schärer (1977)	↓	?	Yes	5	Decidable	yes
Villeneuve et al. (1977)	?	?	Yes	5	Decidable	yes
Wolff et al. (1999)	?	?	No	5	Undecidable	
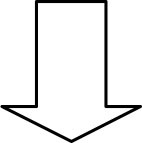						
**In favor of the hypothesis**		**In favor of the alternative hypothesis**			**Undecidable**	
8		0			5	

*Results of hypothesis decision on the basis of the algorithm shown in Figure 2. Studies that confirmed the hypothesis according to the hypothesis-decision algorithm are shaded slightly gray, studies classified as undecidable are shaded dark gray. The algorithm path refers to the numbers on the left side in Figure 2. ↓Significant decrease, n.s., not significant in statistical tests*.

### Descriptive Data Analysis

**Figure 3:**
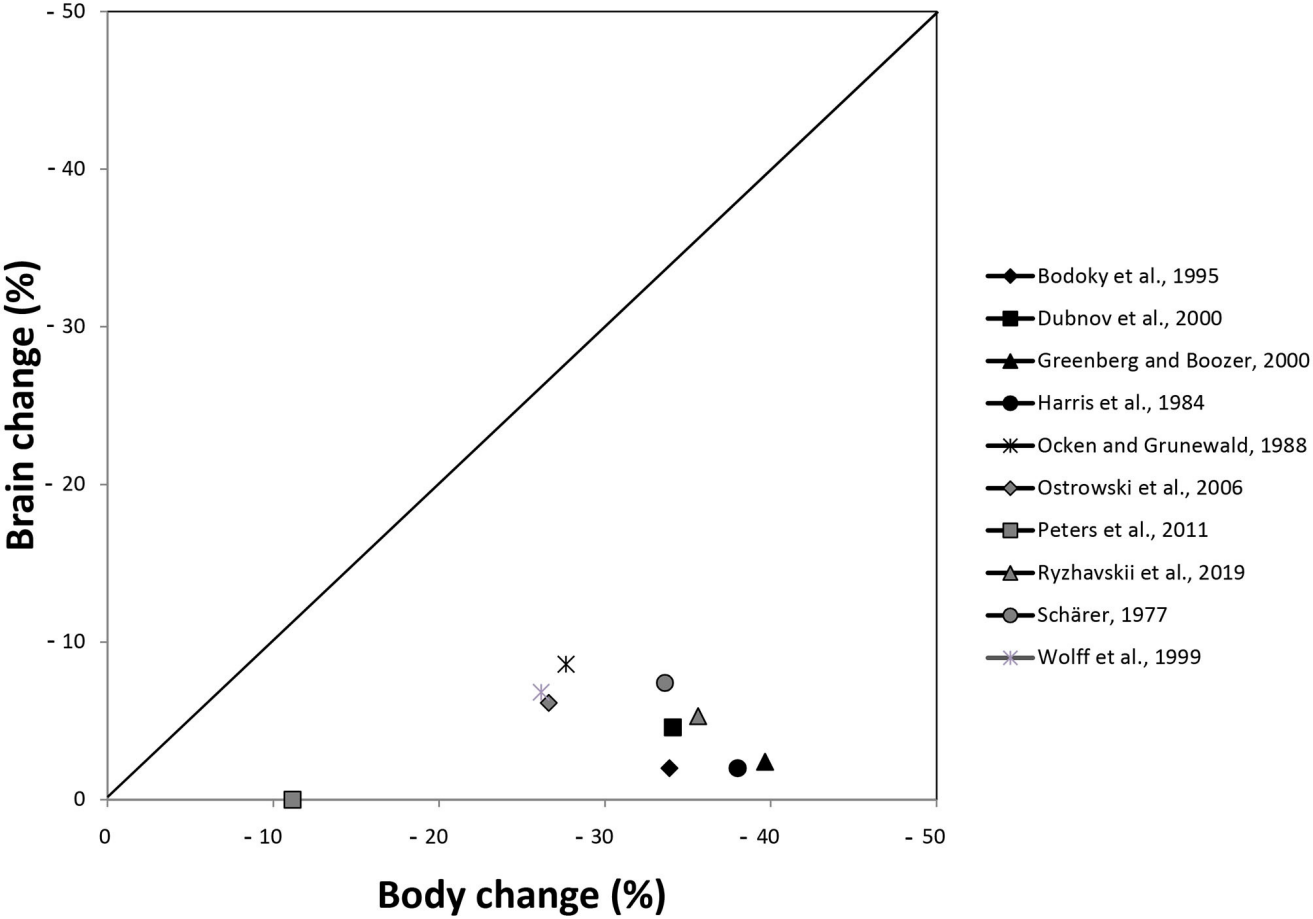
Presentation of percentage changes in brain and body outcomes extracted from Table 1. Harris et al. (1984) conducted their experiment in females and males; here, the data of males are presented. Schärer (1977) conducted three experiments; here, the data of experiment 1 are shown.

Figure 3 shows the percentage changes of brain and body outcomes taken from Table 1. For the inclusion in the graph it was irrelevant whether the hypothesis was decidable or not in a certain study, what was necessary was the availability of sufficient data to calculate the percentage changes. This was the case in 10 studies. In this graphical representation, all 10 studies descriptively showed that the percentage changes in the brain were smaller than the changes in the body.

The 10 studies, which allowed us to calculate percent changes, used interventions ranging from short-term food deprivation to long-term moderate caloric restriction. Because short-term and long-term caloric restriction are known to lead to different physiological pathways (Anderson et al., 2009; Redman and Ravussin, 2009), we asked whether the interventional differences between the 10 studies mattered for brain and body outcomes. Additional descriptive analysis (see Supplementary Information C) does not suggest any pattern according to which the ratio of brain change to body change varies with the duration or intensity of caloric restriction.

## Discussion

A total of 2,804 works was screened by title and abstract, and 232 were analyzed in full text. According to strict selection criteria defined in our PROSPERO pre-announcement and complying with PRISMA guidelines and the predefined hypothesis-decision algorithm, 8 papers were informative enough to decide on the hypothesis (Table 3). All of these 8 decidable papers confirmed the hypothesis, none of them spoke against it, clearly indicating that calorie restriction causes minor mass (energy) changes in the brain as opposed to major changes in the body.

The 8 decidable (and also confirmatory) studies represent a spectrum of diverse experiments which can be summarized as follows. All 8 experiments analyzed here were published between 1977 and 2019, were performed on mice, rats, gazelles, and humans, and the calorie restriction lasted from 4 days to more than 1 year. In animals, ATP and other high-energy phosphates were measured by ^31^P-phosphate-nuclear magnetic resonance (NMR) (Bodoky et al., 1995). Organ weights and total body weight were measured with scales (Schärer, 1977; Villeneuve et al., 1977; Dubnov et al., 2000; Greenberg and Boozer, 2000; Ostrowski et al., 2006; Ryzhavskii et al., 2019). In humans, organ sizes were measured by MRI (Peters et al., 2011). Therefore, in each of these studies, at least one method (NMR, MRI, or scale) was used to measure both brain and body outcomes, allowing for comparison as these outcomes were measured with almost equal precision. Especially for these 8 papers the risk of bias could be rated as low. In the 8 decidable (and confirmatory) studies, brain mass (energy) changes ranged from +0.3 to −7.4%, while body mass (energy) changes ranged from −11.1 to −40.0%.

Multiple redundant mechanisms for safeguarding energy concentrations in the brain have been identified so far. These mechanisms procure the brain with additional fuel when needed: e.g., by increasing heart rate (Jones et al., 2011), visceral fat lipolysis and hepatic ketogenesis (Kubera et al., 2014), muscular lactate release (Qvisth et al., 2008), and by suppressing beta-cell insulin secretion (Hitze et al., 2010). Neuronal ATP can slightly decrease when the brain is undersupplied (e.g., due to lack of food) or when the brain needs more energy (e.g., due to arousal during stress; Madsen et al., 1995). Particularly in the amygdala (Zhou et al., 2010) and in the ventromedial hypothalamus (VMH) (Miki et al., 2001), reductions in neuronal ATP concentrations are detected by intraneuronal energy sensors (Spanswick et al., 1997; Routh et al., 2014; Toda et al., 2016). Even a small decrease of neuronal ATP, leads to depolarization of VMH neurons through GABAergic disinhibition (Chan et al., 2007), and via glutamatergic mechanisms these VMH neurons activate the sympathoadrenal system (Tong et al., 2007; Lindberg et al., 2013). VMH activation increases blood glucose concentrations and lowers insulin concentrations (Meek et al., 2016; Stanley et al., 2016). These VMH effects on glucose and insulin concentrations are mediated by the SNS and HPA axis, which have been found to vigorously suppress insulin secretion from pancreatic beta-cells (Woods and Porte, 1974; Billaudel and Sutter, 1982; Ahren, 2000; Hitze et al., 2010). At low insulin concentrations, blood glucose can hardly be stored in muscle and fat tissue via the insulin-dependent glucose transporters GLUT-4. Blood glucose can pass the blood-brain barrier more easily via the insulin-independent GLUT-1. The brain needs virtually no insulin to take up glucose (Hom et al., 1984; Hasselbalch et al., 1999; Seaquist et al., 2001). The here described brain protective mechanism is one of the most important and is called cerebral insulin suppression (CIS). In conclusion, GLUT-1 glucose uptake safeguards basal energy supply of vital organs, like brain and immune cells (Deng et al., 2014), while GLUT-4 allows the storage of surplus energy in muscle and fat cells (Shepherd and Kahn, 1999). Thus, cerebral insulin suppression allocates energy to the brain in order to maintain cerebral energy concentrations.

The clinical hallmark of CIS is an inadequate low insulin concentration at a given blood glucose concentration. CIS has been observed in critical conditions like starvation (Byrne et al., 1995), psychological stress (Hitze et al., 2010), myocardial infarction (Taylor et al., 1969), and stroke (Harada et al., 2009). Thus, in cases of food shortage or psychological stress, mechanisms like CIS help to control the intraneuronal ATP concentration within narrow limits (Peters and McEwen, 2015).

Our results fully agree with the predictions of the Selfish Brain theory, but violate the predictions of the gluco-lipostatic theory, of all its variants, and of all other theories that assume a passively supplied brain. We must concede, however, that the more accurate predictions of the Selfish Brain theory come at an increased complexity cost. For more accurate predictions, an energy metabolic model needs to include additional mechanisms such as CIS, which guarantee cerebral energy homeostasis. In this respect we are dealing with a trade-off between the model's accuracy and complexity costs (Gilad-Bachrach et al., 2003).

The evidence presented here may surprise one or the other, leaving them with two options: explain away the evidence or update the basic tenets of energy metabolism. Such an update might replace a brain that is only passively supplied with energy, with a brain that regulates its energy concentrations independently and takes a primary position in a hierarchically organized energy metabolism.

One direction for future systematic reviews could be to investigate whether ischemic stroke, due to interrupted blood-to-brain energy flow, causes major changes in the body. Given that the brain regulates its own energy content, body changes as hyperglycemia or weight loss are predicted. The subsequent question would be whether these changes are large enough to be clinically relevant. Another direction for further development could be to study the outcomes for brain and body when the energy flow from blood to muscle or fat is interrupted. This is the case with untreated diabetes mellitus type 1. Given that the brain self-regulates its energy content with highest priority, it is predicted that the severe hyperglycemia in untreated type 1 diabetes mellitus affects the brain high-energy-phosphate concentrations only to a limited extent.

## Data Availability Statement

The data analyzed in this study is subject to the following licenses/restrictions: systematic review. Requests to access these datasets should be directed to Achim Peters, achim.peters@uksh.de.

## Author Contributions

MS developed the search strategies that BK and AP approved. MS screened the article titles or abstracts against the inclusion and exclusion criteria. BK checked this step and disagreements were resolved where necessary by consulting the third reviewer AP. MS and BK independently analyzed the full text and disagreements were resolved where necessary by consultation with the third reviewer AP. MS extracted the data, which BK and AP independently checked. MS assessed the risk of bias, which BK and AP independently checked and approved. AP developed the hypothesis-decision algorithm, which MS and BK agreed upon. MS and BK independently conducted the hypothesis decision, which AP approved. BK and AP wrote the manuscript. All authors contributed to manuscript revision, read, and approved the submitted version.

## Conflict of Interest

The authors declare that the research was conducted in the absence of any commercial or financial relationships that could be construed as a potential conflict of interest.

## References

[B1] Ahren B. (2000). Autonomic regulation of islet hormone secretion-implications for health and disease. Diabetologia 43, 393–410. 10.1007/s001250051322 10819232

[B2] Anderson R. M. Shanmuganayagam D. Weindruch R. (2009). Caloric restriction and aging: studies in mice and monkeys. Toxicol. Pathol. 37, 47–51. 10.1177/0192623308329476 19075044 PMC3734859

[B3] Billaudel B. Sutter B. C. (1982). Immediate *in-vivo* effect of corticosterone on glucose-induced insulin secretion in the rat. J. Endocrinol. 95, 315–320. 10.1677/joe.0.0950315 6757363

[B4] Bodoky G. Yang Z. J. Meguid M. M. Laviano A. Szeverenyi N. (1995). Effects of fasting, intermittent feeding, or continuous parenteral nutrition on rat liver and brain energy metabolism as assessed by 31P-NMR. Physiol. Behav. 58, 521–527. 10.1016/0031-9384(95)00078-W 8587960

[B5] Byrne M. M. Sturis J. Polonsky K. S. (1995). Insulin secretion and clearance during low-dose graded glucose infusion. Am. J. Physiol. 268, E21–E27. 10.1152/ajpendo.1995.268.1.E21 7840177

[B6] Chan O. Lawson M. Zhu W. Beverly J. L. Sherwin R. S. (2007). ATP-sensitive K(+) channels regulate the release of GABA in the ventromedial hypothalamus during hypoglycemia. Diabetes 56, 1120–1126. 10.2337/db06-1102 17251273

[B7] Chaput J. P. Tremblay A. (2009). The glucostatic theory of appetite control and the risk of obesity and diabetes. Int. J. Obes. 33, 46–53. 10.1038/ijo.2008.221 19002144

[B8] Chaudhri O. Small C. Bloom S. (2006). Gastrointestinal hormones regulating appetite. Philos. Trans. R. Soc. Lond., B., Biol. Sci 361, 1187–1209. 10.1098/rstb.2006.1856 16815798 PMC1642697

[B9] Deng D. Xu C. Sun P. Wu J. Yan C. Hu M.. (2014). Crystal structure of the human glucose transporter GLUT1. Nature 510, 121–125. 10.1038/nature13306 24847886

[B10] Dubnov G. Kohen R. Berry E. M. (2000). Diet restriction in mice causes differential tissue responses in total reducing power and antioxidant compounds. Eur. J. Nutr. 39, 18–30. 10.1007/s003940050072 10900554

[B11] Gilad-Bachrach R. Navot A. Tishby N. (2003). An information theoretic tradeoff between complexity and accuracy. Lect. Notes Artif. Int. 2777, 595–609. 10.1007/978-3-540-45167-9_43

[B12] Goodman M. N. Ruderman N. B. (1980). Starvation in the rat. I. Effect of age and obesity on organ weights, RNA, DNA, and protein. Am. J. Physiol. 239, E269–E276. 10.1152/ajpendo.1980.239.4.E269 6158872

[B13] Greenberg J. A. Boozer C. N. (2000). Metabolic mass, metabolic rate, caloric restriction, and aging in male Fischer 344 rats. Mech. Ageing Dev. 113, 37–48. 10.1016/S0047-6374(99)00094-9 10708248

[B14] Harada S. Fujita W. H. Shichi K. Tokuyama S. (2009). The development of glucose intolerance after focal cerebral ischemia participates in subsequent neuronal damage. Brain Res. 1279, 174–181. 10.1016/j.brainres.2009.05.014 19445903

[B15] Harris P. M. Hodgson D. F. Broadhurst R. B. (1984). Response of male and female rats to undernutrition. 1. Changes in energy utilization, body composition and tissue turnover during undernutrition. Br. J. Nutr. 52, 289–306. 10.1079/BJN19840096 6477863

[B16] Hasselbalch S. G. Knudsen G. M. Videbaek C. Pinborg L. H. Schmidt J. F. Holm S.. (1999). No effect of insulin on glucose blood-brain barrier transport and cerebral metabolism in humans. Diabetes 48, 1915–1921. 10.2337/diabetes.48.10.1915 10512354

[B17] Higgins J. P. Altman D. G. Gotzsche P. C. Juni P. Moher D. Oxman A. D.. (2011). The Cochrane collaboration's tool for assessing risk of bias in randomised trials. BMJ 343:d5928. 10.1136/bmj.d5928 22008217 PMC3196245

[B18] Hisatomi Y. Asakura K. Kugino K. Kurokawa M. Asakura T. Nakata K. (2013). Changes in brain tissue and behavior patterns induced by single short-term fasting in mice. PLoS ONE 8:e80085. 10.1371/journal.pone.0080085 24224039 PMC3818263

[B19] Hitze B. Hubold C. van Dyken R. Schlichting K. Lehnert H. Entringer S.. (2010). How the selfish brain organizes its 'supply and demand'. Front. Neuroenerg. 2:7. 10.3389/fnene.2010.00007 20616886 PMC2899523

[B20] Hom F. G. Goodner C. J. Berrie M. A. (1984). A [3H]2-deoxyglucose method for comparing rates of glucose metabolism and insulin responses among rat tissues *in vivo*. Validation of the model and the absence of an insulin effect on brain. Diabetes 33, 141–152. 10.2337/diab.33.2.141 6363168

[B21] Hooijmans C. R. Rovers M. M. de Vries R. B. Leenaars M. Ritskes-Hoitinga M. Langendam M. W. (2014). SYRCLE's risk of bias tool for animal studies. BMC Med. Res. Methodol. 14:43. 10.1186/1471-2288-14-43 24667063 PMC4230647

[B22] Jones A. Steeden J. A. Pruessner J. C. Deanfield J. E. Taylor A. M. Muthurangu V. (2011). Detailed assessment of the hemodynamic response to psychosocial stress using real-time MRI. J. Magn. Reson. Imaging 33, 448–454. 10.1002/jmri.22438 21274988

[B23] Kennedy G. C. (1953). The role of depot fat in the hypothalamic control of food intake in the rat. Proc. R. Soc. Lond. Ser. 140, 578–592. 10.1098/rspb.1953.0009 13027283

[B24] Kubera B. Hubold C. Wischnath H. Zug S. Peters A. (2014). Rise of ketone bodies with psychosocial stress in normal weight men. Psychoneuroendocrinology 45, 43–48. 10.1016/j.psyneuen.2014.03.008 24845175

[B25] Kullmann S. Kleinridders A. Small D. M. Fritsche A. Haring H. U. Preissl H.. (2020). Central nervous pathways of insulin action in the control of metabolism and food intake. Lancet Diabetes Endocrinol. 8, 524–534. 10.1016/S2213-8587(20)30113-3 32445739

[B26] Kuzawa C. W. Blair C. (2019). A hypothesis linking the energy demand of the brain to obesity risk. Proc. Natl. Acad. Sci. U.S.A. 116, 13266–13275. 10.1073/pnas.1816908116 31209026 PMC6612912

[B27] Lindberg D. Chen P. Li C. (2013). Conditional viral tracing reveals that steroidogenic factor 1-positive neurons of the dorsomedial subdivision of the ventromedial hypothalamus project to autonomic centers of the hypothalamus and hindbrain. J. Comp. Neurol. 521, 3167–3190. 10.1002/cne.23338 23696474

[B28] Lowe M. R. Butryn M. L. (2007). Hedonic hunger: a new dimension of appetite? Physiol. Behav. 91, 432–439. 10.1016/j.physbeh.2007.04.006 17531274

[B29] Madsen P. L. Hasselbalch S. G. Hagemann L. P. Olsen K. S. Bulow J. Holm S.. (1995). Persistent resetting of the cerebral oxygen/glucose uptake ratio by brain activation: evidence obtained with the Kety-Schmidt technique. J. Cereb. Blood Flow Metab. 15, 485–491. 10.1038/jcbfm.1995.60 7714007

[B30] Magistretti P. J. Pellerin L. Rothman D. L. Shulman R. G. (1999). Energy on demand. Science 283, 496–497. 10.1126/science.283.5401.496 9988650

[B31] Mayer J. (1953). Glucostatic mechanism of regulation of food intake. N. Engl. J. Med. 249, 13–16. 10.1056/NEJM195307022490104 13063674

[B32] Meek T. H. Nelson J. T. Matsen M. E. Dorfman M. D. Guyenet S. J. Damian V.. (2016). Functional identification of a neurocircuit regulating blood glucose. Proc. Natl. Acad. Sci. U.S.A. 113, E2073–E2082. 10.1073/pnas.1521160113 27001850 PMC4833243

[B33] Miki T. Liss B. Minami K. Shiuchi T. Saraya A. Kashima Y.. (2001). ATP-sensitive K+ channels in the hypothalamus are essential for the maintenance of glucose homeostasis. Nat. Neurosci. 4, 507–512. 10.1038/87455 11319559

[B34] Moher D. Liberati A. Tetzlaff J. Altman D. G. PRISMA Group (2009). Preferred reporting items for systematic reviews and meta-analyses: the PRISMA statement. PLoS Med. 6:e1000097. 10.1371/journal.pmed.1000097 19621072 PMC2707599

[B35] Myers M. G. Cowley M. A. Munzberg H. (2008). Mechanisms of leptin action and leptin resistance. Annu. Rev. Physiol. 70, 537–556. 10.1146/annurev.physiol.70.113006.100707 17937601

[B36] Ocken D. A. Grunewald K. K. (1988). The effects of exercise on catch-up growth of rats recovering from early undernutrition. J. Nutr. 118, 1410–1416. 10.1093/jn/118.11.1410 3273490

[B37] Ostrowski S. Mesochina P. Williams J. B. (2006). Physiological adjustments of sand gazelles (*Gazella subgutturosa*) to a boom-or-bust economy: standard fasting metabolic rate, total evaporative water loss, and changes in the sizes of organs during food and water restriction. Physiol. Biochem. Zool. 79, 810–819. 10.1086/504614 16826507

[B38] Pellerin L. Magistretti P. J. (1994). Glutamate uptake into astrocytes stimulates aerobic glycolysis: a mechanism coupling neuronal activity to glucose utilization. Proc. Natl. Acad. Sci. U.S.A. 91, 10625–10629. 10.1073/pnas.91.22.10625 7938003 PMC45074

[B39] Peters A. Bosy-Westphal A. Kubera B. Langemann D. Goele K. Later W.. (2011). Why doesn't the brain lose weight, when obese people diet? Obes. Facts 4, 151–157. 10.1159/000327676 21577022 PMC6444703

[B40] Peters A. Langemann D. (2009). Build-ups in the supply chain of the brain: on the neuroenergetic cause of obesity and type 2 diabetes mellitus. Front. Neuroenerg. 1:2. 10.3389/neuro.14.002.2009 19584906 PMC2691548

[B41] Peters A. McEwen B. S. (2015). Stress habituation, body shape and cardiovascular mortality. Neurosci. Biobehav. Rev. 56, 139–150. 10.1016/j.neubiorev.2015.07.001 26148986

[B42] Peters A. Schweiger U. Pellerin L. Hubold C. Oltmanns K. M. Conrad M.. (2004). The selfish brain: competition for energy resources. Neurosci. Biobehav. Rev. 28, 143–180. 10.1016/j.neubiorev.2004.03.002 15172762

[B43] Qvisth V. Hagstrom-Toft E. Enoksson S. Bolinder J. (2008). Catecholamine regulation of local lactate production *in vivo* in skeletal muscle and adipose tissue: role of -adrenoreceptor subtypes. J. Clin. Endocrinol. Metab. 93, 240–246. 10.1210/jc.2007-1313 17986640

[B44] Redman L. M. Ravussin E. (2009). Endocrine alterations in response to calorie restriction in humans. Mol. Cell. Endocrinol. 299, 129–136. 10.1016/j.mce.2008.10.014 19007855 PMC3856718

[B45] Routh V. H. Hao L. Santiago A. M. Sheng Z. Zhou C. (2014). Hypothalamic glucose sensing: making ends meet. Front. Syst. Neurosci. 8:236. 10.3389/fnsys.2014.00236 25540613 PMC4261699

[B46] Ryzhavskii B. Y. Lebed'ko O. A. Lazinskaya O. V. (2019). Immediate and delayed effects of food restriction on some parameters of brain development in rats. Bull. Exp. Biol. Med. 167, 104–110. 10.1007/s10517-019-04471-7 31177452

[B47] Schärer K. (1977). The effect of chronic underfeeding on organ weights of rats. How to interpret organ weight changes in cases of marked growth retardation in toxicity tests? Toxicology 7, 45–56. 10.1016/0300-483X(77)90037-3 841584

[B48] Schwartz M. W. Seeley R. J. Zeltser L. M. Drewnowski A. Ravussin E. Redman L. M.. (2017). Obesity pathogenesis: an endocrine society scientific statement. Endocr. Rev. 38, 267–296. 10.1210/er.2017-00111 28898979 PMC5546881

[B49] Seaquist E. R. Damberg G. S. Tkac I. Gruetter R. (2001). The effect of insulin on *in vivo* cerebral glucose concentrations and rates of glucose transport/metabolism in humans. Diabetes 50, 2203–2209. 10.2337/diabetes.50.10.2203 11574399

[B50] Shepherd P. R. Kahn B. B. (1999). Glucose transporters and insulin action-implications for insulin resistance and diabetes mellitus. N. Engl. J. Med. 341, 248–257. 10.1056/NEJM199907223410406 10413738

[B51] Spanswick D. Smith M. A. Groppi V. E. Logan S. D. Ashford M. L. (1997). Leptin inhibits hypothalamic neurons by activation of ATP-sensitive potassium channels. Nature 390, 521–525. 10.1038/37379 9394003

[B52] Stanley S. A. Kelly L. Latcha K. N. Schmidt S. F. Yu X. Nectow A. R.. (2016). Bidirectional electromagnetic control of the hypothalamus regulates feeding and metabolism. Nature 531, 647–650. 10.1038/nature17183 27007848 PMC4894494

[B53] Taylor S. H. Saxton C. Majid P. A. Dykes J. R. Ghosh P. Stoker J. B. (1969). Insulin secretion following myocardial infarction with particular respect to the pathogenesis of cardiogenic shock. Lancet 2, 1373–1378. 10.1016/S0140-6736(69)90928-3 4188271

[B54] Toda C. Kim J. D. Impellizzeri D. Cuzzocrea S. Liu Z. W. Diano S. (2016). UCP2 regulates mitochondrial fission and ventromedial nucleus control of glucose responsiveness. Cell 164, 872–883. 10.1016/j.cell.2016.02.010 26919426 PMC4770556

[B55] Tong Q. Ye C. McCrimmon R. J. Dhillon H. Choi B. Kramer M. D.. (2007). Synaptic glutamate release by ventromedial hypothalamic neurons is part of the neurocircuitry that prevents hypoglycemia. Cell Metab. 5, 383–393. 10.1016/j.cmet.2007.04.001 17488640 PMC1934926

[B56] Villeneuve D. C. van Logten M. J. den Tonkelaar E. M. Greve P. A. Vos J. G. Speijers G. J.. (1977). Effect of food deprivation on low level hexachlorobenzene exposure in rats. Sci. Total Environ. 8, 179–186. 10.1016/0048-9697(77)90076-6 905821

[B57] Wolff G. L. Kodell R. L. Kaput J. A. Visek W. J. (1999). Caloric restriction abolishes enhanced metabolic efficiency induced by ectopic agouti protein in yellow mice. Proc. Soc. Exp. Biol. Med. 221, 99–104. 10.3181/00379727-221-44390 10352119

[B58] Woods S. C. Porte D. Jr. (1974). Neural control of the endocrine pancreas. Physiol. Rev. 54, 596–619. 10.1152/physrev.1974.54.3.596 4601624

[B59] Zhang Y. Proenca R. Maffei M. Barone M. Leopold L. Friedman J. M. (1994). Positional cloning of the mouse obese gene and its human homologue. Nature 372, 425–432. 10.1038/372425a0 7984236

[B60] Zhou L. Podolsky N. Sang Z. Ding Y. Fan X. Tong Q.. (2010). The medial amygdalar nucleus: a novel glucose-sensing region that modulates the counterregulatory response to hypoglycemia. Diabetes 59, 2646–2652. 10.2337/db09-0995 20627933 PMC3279559

