# Supplementary material for: Brain More Resistant to Energy Restriction Than Body: A Systematic Review
Source: Front Neurosci. 2021 Feb 9;15:639617. doi: 10.3389/fnins.2021.639617 (PMC7900631; doi:10.3389/fnins.2021.639617)
Supplement: Supplementary file 1 [file Data_Sheet_6.PDF]

SUPPLEMENTAL MATERIAL

A: MEDLINE and BIOSIS search strategies

Database 1: MEDLINE via PubMED

#1 Starvation[MH] OR Food Deprivation[MH] OR Fasting[MH] OR Caloric restriction[MH] OR starvation[TI] OR „food deprivation“[TI] OR "food deprived"[TI] OR fasting[TI] OR fasted[TI] OR underfeeding[TI] OR diet[TI] OR diets[TI] OR dieting[TI] OR “low calorie”[TI] OR “low carb”[TI] OR “caloric restriction”[TI] OR “calorie restriction”[TI]

#2 Energy Metabolism[MH] OR ATP[TIAB] OR „Adenosine triphosphate“[TIAB] OR “high energy phosphate”[TIAB] OR “high energy phosphates”[TIAB] OR “energy state”[TIAB] OR “phosphorus magnetic resonance spectroscopy”[TIAB] OR “p magnetic resonance spectroscopy”[TIAB] OR “phosphorus MRS”[TIAB] OR “p MRS” [TIAB] OR mass[TIAB] OR weight[TIAB] OR size[TIAB] OR volume[TIAB] OR“magnetic resonance imaging”[TIAB]

#3 brain[TIAB] OR muscle[TIAB] OR muscles[TIAB] OR “adipose tissue”[TIAB] OR “adipose tissues”[TIAB] OR “fat tissue”[TIAB] OR “fat tissues”[TIAB]

#4 “organ mass”[TIAB] OR “organ size”[TIAB]

#5 (#2 AND #3) OR #4

#6 Animal Experimentation[MH] OR Random Allocation[MH] OR randomized controlled trial[PT] OR clinical study[PT] OR clinical trial[PT] OR clinical trial, veterinary[PT] OR controlled clinical trial[PT] OR randomization[ALL] OR experiment[ALL] OR intervention*[TIAB] OR groups[TIAB] OR randomly[TIAB] OR randomized[TIAB] OR trial[TIAB] OR “animal study”[TIAB] OR “control group”[TIAB]

#7 #1 AND #5 AND #6 NOT “diabetes mellitus”[ALL] NOT review[ALL] NOT supplementation[TIAB] NOT “high-fat diet”[TIAB]

Database 2: Biosis Previews via Biosis Citation Index

#1 TI=("starvation" OR "food deprivation" OR "food deprived" OR “fasting” OR "fasted" OR "underfeeding" OR "diet" OR "diets" OR "dieting" OR "low calorie" OR "low carb" OR "caloric restriction" OR "calorie restriction")

#2 TS=(“energy metabolism” OR “ATP” OR “adenosine triphosphate“ OR “high energy phosphate$” OR “energy state” OR “phosphorus magnetic resonance spectroscopy” OR “phosphorus MRS” OR “p MRS” OR “mass” OR “weight” OR “size” OR “volume” OR “magnetic resonance imaging”)

#3 TS=(“brain” OR “muscle$” OR “adipose tissue$” OR “fat tissue$”)

#4 TS=(“organ mass” OR “organ size”)

#5 (#2 AND #3) OR #4

#6 TS=(“animal experimentation” OR “random allocation” OR “randomized controlled trial” OR “clinical study” OR “clinical trial” OR “randomization” OR “experiment” OR “intervention$” OR “group$” OR “randomly” OR “randomized” OR “trial” OR “animal study” OR “control group”)

#7 #1 AND #5 AND #6 NOT TS=(“diabetes mellitus” OR “review” OR “supplementation” OR “high-fat diet”)

B: Exclusion criteria

Human studies

Humans with known diseases such as:

- Neoplastic Disorders (Tumors of the Nervous System, Pancreatic Cancer, Gastrointestinal Cancer...)
- Hematopoietic Disorders (Hypoproliferative Anemias, Disorders of Hemoglobin, Hemolytic Anemias...)
- Disorders of Hemostasis (Arterial and Venous Trombosis, Coagulation Disorders, Disorders of Platelets...)
- Infectious Diseases (Meningococcal Infections, Tuberculosis, Helminthic Infections...)
- Disorders of the Cardiovascular System (Stroke, Heart Attack, Supraventricular tachycardia...)
- Disorders of the Respiratory System (COPD, Asthma, Bronchiectasis...)
- Disorders of the Kidney and Urinary Tract (Acute Renal Failure, Glomerulonephritis, Kidney Stone...)
- Disorders of the Gastrointestinal System and Liver Diseases (Celiac Disease, Cholestasis, Ulcerative Colitis...)
- Immun-Mediated, Inflammatory and Rheumatologic Disorders (Anaphylaxis, Sarcoidosis, Lupus Erythematodes...)
- Endocrine Disorders (Type 1 Diabetes Mellitus, Type 2 Diabetes Mellitus, Adrenal Insufficiency...)
- Neurologic Disorders (Alzheimer's Disease, Epilepsy, Amyotrophic Lateral Sclerosis...)
- Psychiatric Disorders (Depression, Anorexia nervosa, Anxiety Disorder...)

according to Kasper, D., Fauci, A., Hauser, S., Longo, D., Jameson, J.L., Loscalzo, J. (2015). Harrison's Principles of Internal Medicine, 19th Edition. New York, McGraw-Hill Education.

Animal studies

Animals with known diseases such us:

- Neoplastic Disorders (Tumors of the Nervous System, Pancreatic Cancer, Gastrointestinal Cancer...)
- Hematopoietic Disorders (Hypoproliferative Anemias, Disorders of Hemoglobin, Hemolytic Anemias...)
- Disorders of Hemostasis (Arterial and Venous Trombosis, Coagulation Disorders, Disorders of Platelets...)
- Infectious Diseases (Meningococcal Infections, Tuberculosis, Helminthic Infections...)
- Disorders of the Cardiovascular System (Stroke, Heart Attack, Supraventricular tachycardia...)
- Disorders of the Respiratory System (COPD, Asthma, Bronchiectasis...)
- Disorders of the Kidney and Urinary Tract (Acute Renal Failure, Glomerulonephritis, Kidney Stone...)
- Disorders of the Gastrointestinal System and Liver Diseases (Celiac Disease, Cholestasis, Ulcerative Colitis...)
- Immun-Mediated, Inflammatory, and Rheumatologic Disorders (Anaphylaxis, Sarcoidosis, Lupus Erythematodes...)
- Endocrine Disorders (Type 1 Diabetes Mellitus, Type 2 Diabetes Mellitus, Adrenal Insufficiency...)

- Neurologic Disorders (Alzheimer's Disease, Epilepsy, Amyotrophic Lateral Sclerosis...)
- Psychiatric Disorders (Depression, Anorexia nervosa, Anxiety Disorder...)

according to Kasper, D., Fauci, A., Hauser, S., Longo, D., Jameson, J.L., Loscalzo, J. (2015). Harrison's Principles of Internal Medicine, 19th Edition. New York, McGraw-Hill Education.

<u>Human studies</u>
<u>Humans using medications such as:</u>

- Anti-hypertensive drugs
- CNS (Central Nervous System) medication
- CVD (Cardio vascular disease) medication
- Diabetes medication
- ACE (Angiotensin Converting Enzyme) inhibitors
- Analgesics
- Antidepressant drugs
- Antihistamins
- Antiplatelets
- Antipsychotic drugs
- Anxiolytics
- Beta blocker
- Cortico-steroids
- Diuretics
- Hypnotic drugs
- Lipid lowering drugs
- Nitrate medicine
- Osteoporosis drugs
- Parkinsonism drugs
- Rheumatic athritis drugs
- Thyroid hormones

C: Supplemental figure 1

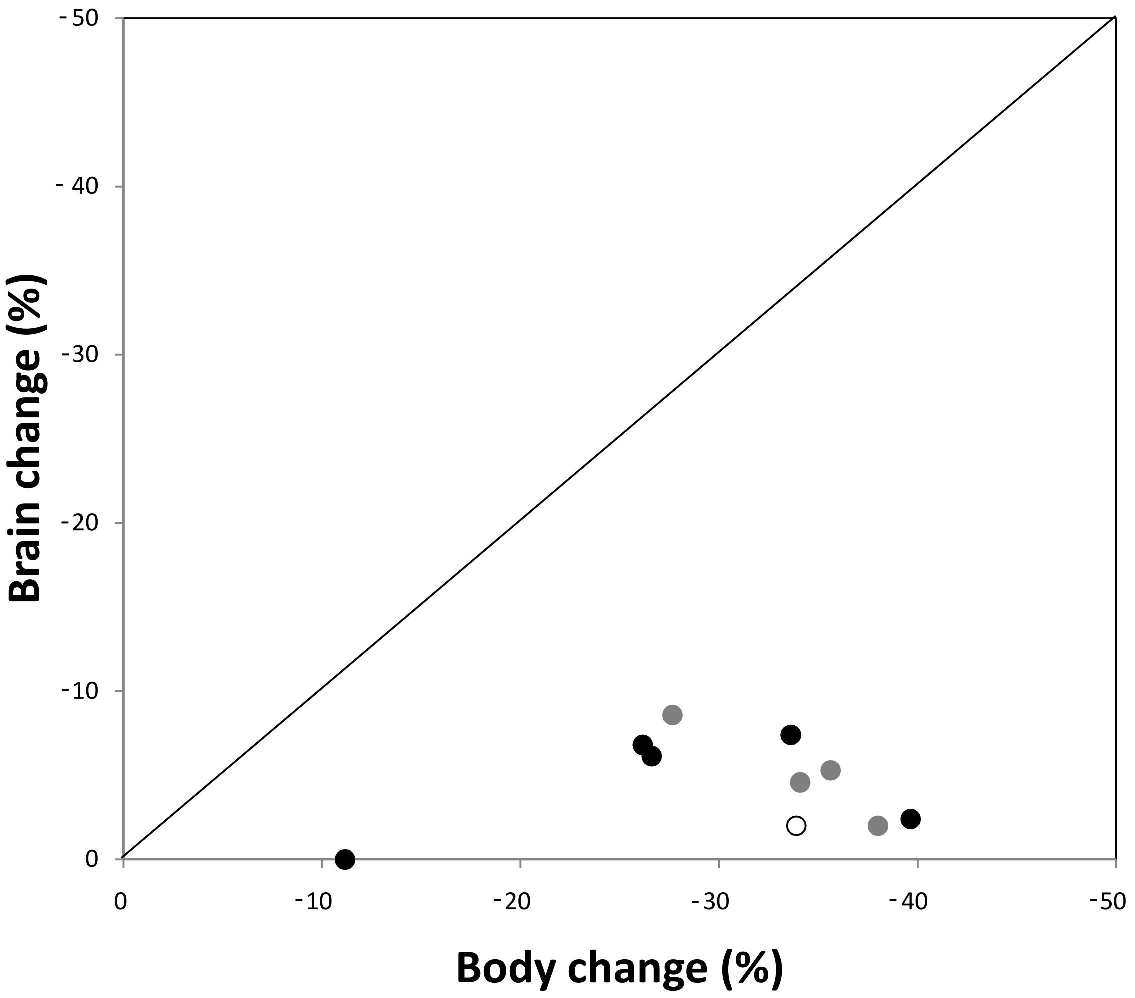

Supplemental figure 1: Presentation of percentage changes in brain and body outcomes according to the duration of the caloric restriction. We used a median split to distinguish between a short (≤40 days) and a long intervention (>40 days). White and grey symbols indicate a shorter caloric restriction, while black symbols indicate a longer caloric restriction. The white symbol refers to the only study that has conducted a complete food deprivation (Bodoky et al., 1995). For further definitons see figure 3.
